# Assessment of renal function in routine care of people living with HIV on ART in a resource-limited setting in urban Zambia

**DOI:** 10.1371/journal.pone.0184766

**Published:** 2017-09-20

**Authors:** Andreas Deckert, Florian Neuhann, Christina Klose, Thomas Bruckner, Claudia Beiersmann, John Haloka, Mannie Nsofwa, Greg Banda, Maik Brune, Helmut Reutter, Dietrich Rothenbacher, Martin Zeier

**Affiliations:** 1 Institute of Public Health, Heidelberg University Hospital, Heidelberg, Germany; 2 Institute of Medical Biometry and Informatics, Heidelberg University Hospital, Heidelberg, Germany; 3 CHRESO University, Lusaka, Zambia; 4 Internal Medicine I and Clinical Chemistry, Heidelberg University Hospital, Heidelberg, Germany; 5 Institute of Epidemiology and Medical Biometry, Ulm University, Ulm, Germany; 6 Division of Nephrology, Heidelberg University Hospital, Heidelberg, Germany; Universidade de Sao Paulo Faculdade de Medicina, BRAZIL

## Abstract

**Introduction:**

Data on renal impairment in sub-Saharan Africa (SSA) remains scarce, determination of renal function is not part of routine assessments. We evaluated renal function and blood pressure in a cohort of people living with HIV (PLWH) on antiretroviral treatment (ART) in the Renal Care Zambia project (ReCaZa).

**Methods:**

Using routine data from an HIV outpatient clinic from 2011–2013, we retrospectively estimated the glomerular filtration rate (eGFR, CKD-Epi formula) of PLWH on ART in Lusaka, Zambia. Data were included if adults had had at least one serum creatinine recorded and had been on ART for a minimum of three months. We investigated the differences in eGFR between ART subgroups with and without tenofovir disproxil fumarate (TDF), and applied multivariable linear models to associate ART and eGFR, adjusted for eGFR before ART initiation.

**Results and discussion:**

Among 1118 PLWH (63,3% female, mean age 41.8 years, 83% ever on TDF; median duration 1461 [range 98 to 4342] days) on ART, 28.3% had an eGFR <90 ml/min, and 5.5% <60 ml/min at their last measurement. Information on other conditions associated with renal impairment was not systematically documented. Fourteen per cent of the PLWH who later switched to TDF-free ART had an initial eGFR lower 60ml/min. Nineteen percent had first-time hypertensive readings at their last visit. The multivariable models suggest that physicians acted according to guidelines and replaced TDF-containing ART if patients developed moderate/severe renal impairment.

**Conclusions:**

Assessment of renal function in SSA remains a challenge. The vast majority of PLWH benefit from long-term ART, including improved renal function. However, approximately 5% of PLWH on ART may have clinically relevant decreased eGFR, and 27% hypertension. While a routine renal assessment might not be feasible, strategies to identify patients at risk are warranted. Targeted monitoring prior and during ART is recommended, however, should not delay ART access.

## Introduction

Zambia, classified as a lower-middle-income country, faces an increase in non-communicable chronic diseases (NCD) such as cardiovascular diseases and others, including chronic kidney disease (CKD) [1]. A longer life expectancy, partly due to antiretroviral therapy (ART), contributes to this transition [2]. HIV prevalence in Zambia is high at 12.4% and the overall ART coverage of people living with HIV (PLWH) is 60% [3].

CKD is considered to be an increasing burden for PLWH [4]. The chronic inflammatory process of HIV infection and the impaired renal function are associated with the development and progression of cardiovascular disease [5]. Currently, the healthcare systems in Sub-Saharan Africa (SSA) cannot adequately cope with CKD and attendant co-morbidities [6].

Epidemiological data about CKD in SSA are scarce, and reliable morbidity and mortality estimates are lacking. Some studies have reported renal insufficiency in up to 33.5% of the Zambian HIV population [7]. Advanced CKD (estimated glomerular filtration rate (eGFR) <60ml/min, or presence of proteinuria) has been estimated to have a prevalence of 13.9% in SSA [4]. Most published CKD data in SSA refer to HIV-infected populations [8–11].

HIV and renal function are closely intertwined; HIV is known to have pathological effects on the kidneys. ART improves renal function in the vast majority of PLWH [12, 13]. Lucas et al. documented that persons with non-suppressed HIV-RNA experienced a rapid decline of GFR over time [14]. In addition, some ART treatment compounds, e.g. tenofovir disoproxile fumarate (TDF), can impair renal function [15–17].

Diagnosis of CKD remains a challenge in resource-limited settings. Serum creatinine determination is the only available CKD marker but not applied an integral part of assessment in many clinical settings and HIV programmes. Despite these limitations, it is important to make informed health decisions based on available data. Hence, analysing data from sites where creatinine determination is performed allows for an estimate of the degree of the problem, in particular for PLWH on ART.

We describe the characteristics of a large HIV cohort (Renal Care Zambia, ReCaZa) on ART at Chreso Ministries (CM) in Lusaka, Zambia. We report results of routinely collected outpatient data and present figures for renal impairment and prevalence of increased blood pressure. Furthermore, we investigate the association between ART and eGFR during follow-up while adjusting for available cofactors.

## Methods

### Ethics statement

The study protocol received ethical clearance from the ethical committee of Heidelberg University (approval number S-024/2014) and Chreso University (approval number 2013-Sept-001). Written informed consent from patients was not required since this retrospective research only used routinely collected data.

### Study site

Since 2004, CM contributes considerably to the provision of HIV services in Lusaka Central and Southern provinces of Zambia. It serves more than 40,000 clients in care, with 15,000 receiving ART. Chreso Ministries in Lusaka (Chreso Lusaka, CL) see the largest number of PLWH. CL is located in a densely populated, low-income area of Lusaka, with estimated 44,000 inhabitants. Patients usually register after voluntary counselling and testing or through maternal and child health services. Following an equitable access approach, persons eligible for ART are 1) PLWH tested positive with a CD4 count <500 cells/ml, 2) HIV+ pregnant or breastfeeding women, 3) partners of these women, 4) individuals with HIV/TB co-infection, and 5) children less than 15 years. After registration, body weight and vital signs (e.g. blood pressure) are recorded. Haemoglobin (Hbg), AST and ALT, serum creatinine and CD4-counts are routinely assessed at enrolment. More specific diagnostic procedures e.g. for diabetes or viral hepatitis are not part of the routine assessment.

### Study population, data collection and management

Starting with the first patient file that came into the data management unit on a clinic day, and as many of the consecutive files as possible were entered into a separate study database. Data were included in the analysis if the persons were 18 years or older at HIV diagnosis, had at least one creatinine determination between January 1, 2011 and December 31, 2013, and if they had received ART for at least 90 days at their last creatinine measurement. If ART was initiated before HIV diagnosis (persons transferred in), their HIV diagnosis date was set to the initiation of ART. Data were processed, transferred and analysed in a pseudonymized form.

### Laboratory measurements

Samples were obtained onsite by trained lab technicians as part of a routine evaluation. Plasma creatinine concentrations were determined by photometric assay (Jaffe reaction, Roche). Reference values were 62–106 μmol/l for men and 44–80 for women. Standardized control samples were analysed every day. Repetitive control measurements with standard samples in the normal and pathological range resulted in inter-assay coefficients of variation of 5.5% and 3.7%, respectively. Complete blood count including hemoglobin concentration (ABX Micros 60, Axonlab, Switzerland), CD4 count (BD Facscount, USA), and alanine transaminase (ALT) and aspartate transaminase (AST) analysis (Cobras c111, Roche, Germany) were performed.

### Estimation of GFR and categorization of renal impairment

We estimated the eGFR according to the CKD-EPI equation [18]:

$$eGFR=141\times {min(\frac{S_{Crea}}{\kappa },1)}^{\alpha }\times {max(\frac{S_{Crea}}{\kappa },1)}^{-1.209}\times 0.993^{age}\times 1.018_{\left[if\;female\right]}$$

(κ = 0.7 for women, κ = 0.9 for men; α = -0.329 for women, α = -0.411 for men).

Persons were classified in eGFR categories 1 to 5, according to the Kidney Disease Improving Global Outcomes (KDIGO). Persons with an eGFR equal to or above category 3a (< 60ml/min) were categorized as having a renal impairment requiring immediate attention.

### Blood pressure

Blood pressure (BP) was taken twice using manual syphingo-manometers, with the patients seated with their arm resting on a desk. Persons were grouped into systolic BP <120, 120–139, and ≥140 mm HG categories. Mean arterial pressure (MAP = diastolic+1/3[systolic-diastolic]) was calculated.

### Statistical analysis

Data were retrieved for 1) date of first laboratory test after HIV diagnosis, and 2) date of last creatinine determination between 2011 and 2013. Statistical comparison was done by means of Wilcoxon signed rank test. Univariate analyses at time of last creatinine determination were used to investigate differences between persons continuously receiving TDF-free ART (ART_TDF free_), continuously receiving TDF-containing ART (ART_TDF_), and persons whose TDF-containing ART was switched to a TDF-free ART (ART_swtiched_) over the course of time.

A multivariable linear regression model was applied to the subgroup of patients with an additional creatinine reading available after HIV diagnosis but before ART initiation (N = 565) to investigate associations of eGFR and ART categories at their last measurement, adjusted for initial eGFR, age and sex. After confounder assessment, systolic blood pressure and length of HIV duration were included. ART time period was highly correlated with HIV duration time (Pearson correlation coefficient 0.92), thus excluded from the model. Additionally, two sensitivity analyses at the last point of measurement were performed (N = 1118) to account for possible selection bias in the subgroup, and to investigate differences in single ART regimens. Treatment interruptions and crossovers could not be determined. All modelling was done in SAS version 9.3. Linear relations of all continuous covariates to the outcome were confirmed by the multivariate fractional polynomials approach in Stata version 14 [19].

## Results

### Recruitment

**Fig 1:**
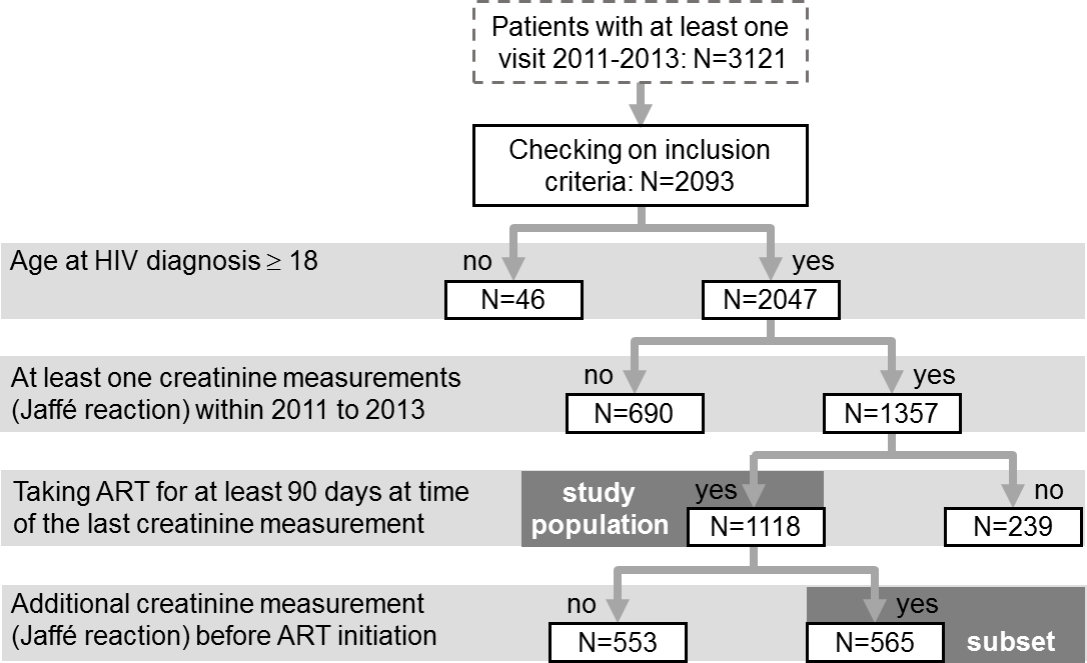
Study population flowchart.

CL recorded 3121 PLWH with at least one visit between January 1, 2011 and December 31, 2013. We were able to screen 2093 patient records with respect to the inclusion criteria, according to the procedure described above. Out of these, 1118 (53.4%) patient records contained at least one creatinine measurement and these patients had received ART for at least 90 days, thus forming the study population (anonymized dataset see S1 Table). A subset of 565 (27,0%) persons had an additional creatinine measurement before ART initiation (see flowchart Fig 1). The majority of patients were women (63.3%). Median age at HIV diagnosis was 37 years. There were 66 persons who had been transferred in and who had initiated ART prior to registration at CL and whose HIV diagnosis was then set to the date of treatment initiation. Among the female enrolees no case of short term ART for PMTCT was recorded.

### Differences between first and last measurement

All health related parameters (except AST) differed significantly between initial and last measurement (Table 1). Most parameters were measured first time right after HIV confirmation; mean HIV duration was 80.2 days. Due to historically incomplete data at time of ART coverage programme start, initial creatinine (i.e. before ART initiation) was not available in 49.5%. However, those with missing creatinine had similar sex (p = 0.98), age (p = 0.67), and Hgb (p = 0.34) compared to those considered in the multivariable model with baseline adjustment. In contrast, CD4 count (available in 58.4% versus 94.9%) was statistically significantly (203 μl-1 versus 266 μl-1; p<0.001) and BMI slightly higher (22.1 kg/m2 versus 22.7 kg/m2; p = 0.02) in the latter. Initially, 62.8% of the patients presented with a CD4 count less than 200 cells/μl, in contrast to 32.6% at last measurement. In the group with creatinine measured before ART initiation, eGFR decreased from median 108.3 to 103.3 ml/min/1.73 m2 from nearest to onsite HIV testing to last measurement (p<0.001). Around one quarter of the PLWH on ART had any degree of impaired renal function at first measurement (24.6%, 95% confidence interval (CI) [21.1; 28.2], N = 565), and 5.0% (CI [3.2; 6.7]) eGFR<60ml/min, compared to 28.3% (CI [25.7; 30.9], N = 1118), and 5.5% (CI [4.2; 6.9]), respectively, at last measurement. Initially, 12% were hypertensive (15.4% missing), compared to 27% at last measurement (17.7% missing).

**Table 1:** HIV and renal impairment related variables at two specific points in time of HIV patients on ART at Chreso Ministries in Lusaka, Zambia, between 2011–2013 and KDGIO eGFR category classification.

		**nearest to onsite HIV testing** ^**a**^				**last creatinine measurement**				
**variable**		**N**	**%**	**median**	**(Q1; Q3)**	**N**	**%**	**median**	**(Q1; Q3)**	**p-value** ^**b**^
duration of HIV [days]		1118	100	1	(0; 4)	1118	100	1642	(1001; 2223)	<0.001
ART duration [days]						1118	100	1461	(768; 2103)	
age [years]		1118	100	37	(31; 43)	1118	100	41	(35; 47)	
BMI [kg/m^2^]	men	333	81	20.9	(19.0; 23.4)	337	82	22.7	(20.2; 25.5)	<0.001
	women	592	84	22.5	(19.9; 25.2)	591	83	24.9	(21.6; 28.4)	<0.001
mean arterial pressure [mmHg]		945	85	86.7	(80.0; 96.7)	920	82	94.3	(84.7; 105.7)	<0.001
systolic blood pressure [mmHg]		946	85	112	(107; 127)	920	82	125	(114; 141)	<0.001
CD4 [ml^-1^]		859	77	194	(103; 302)	877	78	392	(264; 530)	<0.001
haemoglobin [g/dl]		769	69	11.7	(10.1; 13.2)	788	70	12.9	(11.6; 14.1)	<0.001
AST [U/l]		580	52	27.3	(19.6; 37.8)	993	89	27.2	(22.0; 34.0)	0.46
ALT [U/l]		586	52	17.6	(11.0; 27.5)	1051	94	24.2	(17.7; 33.0)	<0.001
eGFR (CKD-EPI) [ml/min/1.73m^2^]		565	51	108.3	(88.8; 118.5)	1118	100	103.3	(86.9; 114.0)	<0.001
**eGFR category** ^**b**^		**565**	**100**			**1118**	**100**			
1) eGFR >=90 [l/min]: normal/high glomerular filtration rate		426	75.4			802	71.7			
2) eGFR 60–89: mildly decreased kidney function		111	19.7			254	22.7			
3a) eGFR 45–59: mildly to moderately decreased		10	1.8	^c^		42	3.8	^d^		
3b) eGFR 30–44: moderately to severely impaired		9	1.6	^c^		12	1.1	^d^		
4) eGFR 15–29: severely decreased kidney function		4	0.7	^c^		6	0.5	^d^		
5) eGFR <15: kidney failure		5	0.9	^c^		2	0.2	^d^		
Missing		553	49.5			0	0			

^a^ eGFR values restricted to measurements after HIV diagnosis but before ART initiation

^b^ Wilcoxon signed rank test p-value for paired comparison of first and last value

^c^ eGFR categories 3a) to 5) add up to 5.0%

^d^ eGFR categories 3a) to 5) add up to 5.5%

### Differences in the ART groups

**Fig 2:**
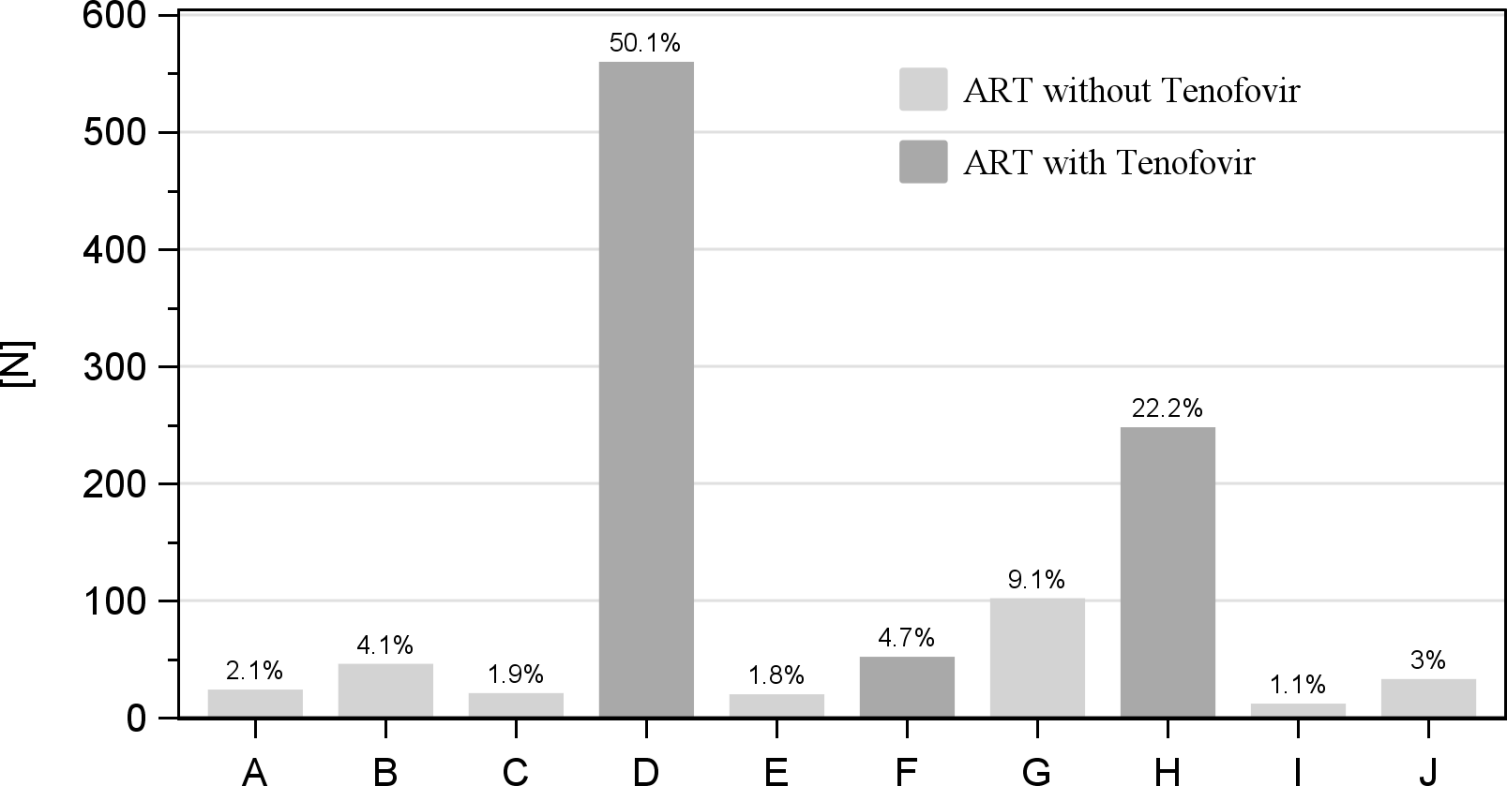
ARTs regimen as documented at time of last creatinine measurement. A = Stavudine (d4T), 3TC, Nevirapine (NVP); B = AZT, 3TC, Efavirenz (EFV); C = Abacavir (ABC), 3TC, EFV; D = TDF, Emtricitabine (FTC), EFV; E = ABC, Lopinavir (LPV), Ritonavir (rtv); F = TDF, FTC, LPV/rtv; G = AZT, 3TC, NVP; H = TDF, FTC, NVP; I = ABC, 3TC, NVP; J = not classifiable/other combinations.

In the total of 1118 persons, 929 (83.1%) had ever received TDF containing ART, 73 of those had switched to TDF-free ART, and 2 vice versa. The combination TDF/Emtricitabine/Efavirenz was most frequently applied (50.1%; see Fig 2). In 3.1% of the patients the ART regimen could not clearly be determined. However, adherence to ART seemed to be high, vast majority of patients continuously received ART according to the patient records (see S1 Fig). ART_TDF_ subjects presented with the shortest HIV and ART durations, as well as the lowest MAP and systolic BPs, whereas their AST was the highest (Table 2). ART_switched_ cases ultimately had lower CD4 counts and significantly higher creatinine, thus pointing to possible cases of HIV nephropathy.

**Table 2:** Differences at last measurement between HIV patients who had never received TDF-containing ART(ART_TDF free_), those who continuously took a TDF-containing ART (ART_TDF_), and those who switched to TDF free (ART_switched_) at Chreso Ministries in Lusaka, Zambia 2011–2013.

		ART_TDF free_ (N = 189)				ART_TDF_ (N = 856)				ART_switched_ (N = 73)				
variable		N	%	mean	sd	N	%	mean	sd	N	%	mean	sd	p-value
Sex	women	124	66			533	62			51	70			0.34^a^
duration of HIV infection [days]		189	100	2055	803	856	100	1511	789	73	100	1776	844	<0.001^b^
duration of ART intake [days]		189	100	1918	713	856	100	1357	791	73	100	1635	782	<0.001^b^
Age [years]		189	100	42.2	9.1	856	100	41.6	8.9	73	100	42.9	11.3	0.42^b^
BMI [kg/m^2^]	men	48	74	23.3	4.2	271	84	23.1	4.2	18	82	24.4	4.3	0.45^b^
	women	104	84	25.3	4.9	447	81	25.5	5.1	40	78	23.6	3.9	0.06^b^
mean arterial pressure [mmHg]		149	79	100.0	15.7	711	83	95.5	16.2	60	82	98.1	18.7	0.006^b^
systolic blood pressure [mmHg]		149	79	134.3	22.1	711	83	127.1	22.1	60	82	131.9	26.1	<0.001^b^
CD4 [ml^-1^]		147	78	455	251	689	80	407	203	41	56	404	247	0.05^b^
haemoglobin [g/dl]		137	72	13.0	1.8	609	71	12.7	2.0	42	58	12.0	2.1	0.02^b^
AST [U/l]		161	85	28.6	15.2	779	91	31.0	18.1	53	73	25.8	9.6	0.04^b^
ALT [U/l]		173	92	28.0	25.5	821	96	28.9	18.7	57	78	24.7	14.8	0.28^b^
creatinine [mg/dl]		189	100	0.8	0.3	856	100	0.8	0.3	73	100	1.1	0.6	<0.001^b^
CKD-EPI eGFR [ml/min/1.73m^2^]		189	100	100.8	21.4	856	100	101.1	21.4	73	100	81.5	30.4	<0.001^b^
eGFR category	1, 2	178	94			826	96			52	71			<0.001^a^
	3a-5	11	6			30	4			21	29			
Anaemia^c^	yes	44	32			222	36			20	48			0.19^a^
	no	93	68			387	64			22	52			
	missing	52	28			247	29			31	42			
liver encymes	yes	4	3			16	2			1	2			0.94^a^
>2•ULN^d^	no	157	97			760	98			52	98			
	missing	28	15			80	9			20	27			
liver encymes	yes	1	1			0	0			0	0			0.08^a^
>5•ULN^e^	no	159	99			775	100			53	100			
	missing	29	15			81	9			20	27			
systolic blood	<120 mmHg	40	27			284	40			22	37			0.004^a^
pressure	120–139 mmHg	56	38			250	35			16	27			
	> = 140 mmHg	53	36			177	25			22	38			
	missing	40	21			145	17			13	18			

^a^ χ2-test

^b^ ANOVA, two-sided

^c^ Anaemia was defined as Hgb below 13 [g/dl] for men, and below 12 [g/dl] for women.

^d^ Liver disease was defined if at least ALT or AST were twice the upper limit of normal (ULN) of 50 [U/l] for men and 35 [U/l] for women.

^e^ Presence of severe liver disease was assumed if at least ALT or AST were five times higher than the ULN.

PLWH receiving ART_TDF_ showed less frequent high blood pressure (Table 2), and less frequent moderate/severe eGFR impairment at the second assessment. Initially, 11.0% of ART_TDF_, 10.1% of ART_switched_, and 7.7% of ART_TDF free_ had hypertensive BP readings (> = 140 mmHg). In the ARTswitched group, 28.8% ultimately suffered from moderate/severe renal impairment. ART_TDF free_ patients had a median eGFR decrease of -3.0 l/min/1.73 m2, ART_TDF_ -6.5, and ART_switched_ -8.5 over the course of observation (unadjusted).

### Multivariable analyses

The multivariate linear regression model yielded a slightly more favourable profile for ART_TDF free_ subjects (Table 3), with the ART_switched_ group having the lowest eGFR. Although not adjusted for initial eGFR, the first sensitivity analyses resulted in similar ART group effect sizes. In the second sensitivity analyses, all estimates were lower compared to the largest group of a TDF-containing regimen. The strongest effect was found in an abacavir combination therapy, applied in only 1.9% of patients. The second strongest effect was visible in a TDF-containing regimen, applied in 4.6%. Dichotomizing eGFR in logistic regression with a threshold of 60 l/min confirmed these results (ART_TDF free_ OR = 1.1, CI [0.5; 2.6]; ART_switched_ OR = 9.6, CI [4.8; 19.5]). A threshold of 90 l/min yielded ART_TDF free_ OR = 0.8 (CI [0.5; 1.3]), and ART_switched_ OR = 3.3 (CI [1.8, 5.9]).

**Table 3:** Multivariate linear regression models: i) adjusted for initial eGFR, age, sex, time of HIV duration, plus three ART categories; ii) adjusted for ART categories at last measurement (sensitivity analysis); iii) adjusted for specific ART regimen at last measurement (sensitivity analysis).

		model i)^a^				model ii)^b^				model iii)^c^		
variable	%^§^	estimate	se	Pr > F	%^§^	estimate	se	Pr > F	%^§^	estimate	se	Pr > F
Intercept		121.3	9.73	<0.0001		155.3	4.64	<0.0001		154.8	4.64	<0.0001
Sex		0.41	2.22	0.85		-2.79	1.44	0.05		-2.48	1.52	0.10
Age		-0.69	0.14	<0.0001		-0.96	0.08	<0.0001		-0.93	0.08	<0.0001
Initial eGFR [l/min]		0.15	0.04	0.0004		excluded				excluded		
systolic blood pressure [mmHg]		-0.05	0.05	0.31		-0.09	0.03	0.004		-0.09	0.03	0.005
duration HIV infection [days]		-0.002	0.001	0.20		-0.0007	0.0009	0.43		-0.0003	0.0009	0.77
TDF_ART	78.2	reference			76.6	reference						
TDF_free_ART	15.4	1.92	3.06	0.53	16.9	1.61	1.92	0.40				
TDF_switched_ART	6.4	-15.36	4.17	0.0003	6.5	-16.69	2.78	<0.0001				
A) d4T, 3TC, NVP^e^									2.1	-0.46	4.99	0.93
B) AZT, 3TC, EFV^e^									4.1	-0.53	3.66	0.89
C) ABC, 3TC, EFV^e^									1.9	-25.31	5.37	<0.0001
D) TDF, FTC, EFV^d^^,^ ^e^									50.1	reference		
E) ABC, LPV, rtv^e^									1.8	-10.36	4.83	0.03
F) TDF, FTC, LPV/rtv ^d^^,^ ^e^									4.6	-12.95	3.39	0.0001
G) AZT, 3TC, NVP^e^									9.1	-0.05	2.55	0.98
H) TDF, FTC, NVP^d,^ ^e^									22.2	-2.57	1.81	0.16
I) ABC, 3TC, NVP^e^									1.1	-20.49	6.26	0.001
J) not classifiable/other combinations^e^									3.0	-10.99	4.20	0.009

^a^ Linear regression model, adjusted for initial eGFR; N = 457 observations used; R^2^ = 18.7%

^b^ Sensitivity analyses, at least one eGFR between January 1, 2011 and December 31, 2013; N = 919 observations used; R^2^ = 22.1%.

^c^ Sensitivity analyses, at least one eGFR between January 1, 2011 and December 31, 2013, specific ART regimens; N = 919 observations used; R^2^ = 23.3%

^d^ Tenofovir containing regimen

^e^ ABC = abacavir, TDF = tenofovir disoproxil fumarate, d4T = stavudine, EFV = efavirenz, FTC = emitricitabine, LPV = liponavir, rtv = ritonavir, NVP = nevirapine

^§^ percentage of ART regimen application at last measurement

### Trend analyses

Confined to those with a creatinine reading before ART initiation, initially approximately 14% of ART_switched_ individuals presented with an eGFR<60ml/min, compared to 3.2% of ART_TDF_, and 10.3% of ART_TDF free_. At last measurement, the eGFR was <60ml/min in 33.3% of the ART_switched_ group, compared to 4.1% in ART_TDF_, and 5.7% of the ART_TDF free_ (Table 4).

**Table 4:** Initial eGFR categories and those at time of last creatinine measurement, separated by changes in ART regimen (N = 565).

	Initial eGFR category^a^						eGFR category at last measurement^b^					
eGFR	ART_switched_		ART_TDF_		ART_TDF free_		ART_switched_		ART_TDF_		ART_TDF free_	
category	N	(%)	N	(%)	N	(%)	N	(%)	N	(%)	N	(%)
1	17	(47.2)	345	(78.0)	64	(73.6)	14	(38.9)	314	(71.0)	65	(74.7)
2	14	(38.9)	83	(18.8)	14	(16.1)	10	(27.8)	110	(24.9)	17	(19.5)
3a	1	(2.8)	7	(1.6)	2	(2.3)	7	(19.4)	14	(3.2)	4	(4.6)
3b	3	(8.3)	3	(0.7)	3	(3.4)	3	(8.3)	2	(0.5)	0	(0.0)
4	0	(0.0)	1	(0.2)	3	(3.4)	1	(2.8)	1	(0.2)	1	(1.1)
5	1	(2.8)	3	(0.7)	1	(1.2)	1	(2.8)	1	(0.2)	0	(0.0)

^a^ χ2-test p-value 0.0001

^b^ χ2-test p-value <0.0001

Patients with an initial eGFR >60ml/min presented lower eGFR at last measurement (ART_TDF free_ median -5.8, ART_TDF_ -7.8, ART_switched_ -11.6ml/min; Pr>χ^2^ 0.7 Kruskal-Wallis test; unadjusted). In contrast, the majority of persons with an initial eGFR <60ml/min maintained or re-gained glomerular filtration during ART; whereas the eGFR of two individuals (7.1%) further deteriorated in at least one category, while the eGFR of N = 23 (82.1%) improved.

A total of 22.6% of initially non-hypertensive ART_TDF free_ (42.7% missing), 28.0% of ART_switched_ (21.9% missing), and 17.8% of ART_TDF_ (28.6% missing) had high blood pressure readings at the second assessment. Blood pressure in patients with initial eGFR <60ml/min (N = 20 with two BP readings) did not improve; in contrast, around 40% of those with initially normal blood pressure were found with hypertensive readings later.

## Discussion

This retrospective analysis based on routine clinical data of PLWH on ART is in line with similar studies, thus strengthens the evidence regarding renal impairment among PLWH in SSA, and in particular in urban Zambia. Despite of the limitations, the results indicate the existence of a considerable problem of impaired renal function in PLWH in SSA.

Compared to Mulenga et al., who used data from the Zambian HIV program in 2007, our renal impairment estimates appear higher (eGFR <60ml/min: with TDF (initial ART_TDF_ plus ART_switched_) 3.9% versus 1.9%, without TDF 8.8% versus 4.0%) [20]. The Mulenga cohort was younger, had a lower BMI and median CD4 counts of 151/ml in ART_TDF_ and 172/μl in ART_TDF free_ starters compared to our overall median of 194/μl. Around 62% started ART_TDF_, compared to 83% in our cohort. Additionally, the differences could be partly explained by our use of the CKD-Epi formula without adjustment for persons of African origin. The factor for persons of African origin derives from studies in the US and might be of value in the US to adjust for socio-economic status, but does not seem to be applicable in Africa [18]. The equation also performs better in persons with mildly impaired kidney function compared to other formulas, hence qualifies as a screening tool to assess onset of CKD in resource-limited settings [18]. However, Glaser et al. found that the creatinine CKD-Epi seems to overestimate eGFR in PLWH compared to CystatinC formulas. Consequently, we assume that our data rather underestimate the extent of renal impairment.

Despite missing values, we could observe that the eGFR category of individuals with initially moderate/severe renal impairment either remained constant or improved on ART (N = 25, 89.3%), regardless of the chosen regimen. This supports the assumption by Mulenga et al. that even potentially nephrotoxic ART regimens can improve pre-existing HIV-associated nephropathy [20]. The eGFR detoriation in the ART_switched_ group could be a consequence of some patients referred to a different regimen after diagnosis of CKD.

Changes in the most frequently prescribed ART over time reflect changes in recommendation in the national guidelines: Most TDF-free patients started ART before 2008 (median 2007) and medication was maintained. Some of the first persons receiving TDF containing ART might have already developed impaired eGFR/CKD, thus medication was switched (median 2008). The median initiation year of those always on TDF was 2009, when TDF had become part of standard first line treatment.

Laprise et al. reported a significant but mild eGFR loss attributable to long-term TDF exposure [21]. This finding was similar to results from Morcroft et al. regarding TDF and Ritonavir boosted Lopinavir [22]. However, we also saw a decline associated with ART containing Abacavir (small number of individuals). Furthermore, in a study from Brazil, the loss in eGFR (CKD-EPI) was approximately 10 ml/min over a period of three years, and male sex, systolic arterial hypertension and age >50 were associated with that decline [23]. This finding could be of importance in aging African cohorts. In our multivariable analysis individuals always receiving TDF showed only a slight, but not significant, eGFR reduction. The group of PLWH who changed to a TDF-free regimen had the lowest eGFR. Our observations suggest that clinicians reacted according to guidelines and replaced TDF-containing ART if moderate/severe renal impairment developed during the course of treatment, which is also reflected by the lower frequency of severe/moderate renal impairment in the ART_TDF_ group (survival bias). In contrast, in persons with renal impairment present already initially, renal function might not be related to a nephrotoxic drug effect rather to a variety of potential causes (e.g. inflammation, diabetes, blood pressure, etc.).

This study is descriptive and does not allow to draw conclusions about causality. In particular, it was frequently not possible to allocate data of former regimen changes to accurate time periods. Furthermore, we could not define the potential onset of impaired renal function and clearly relate it to ART. In addition, no systematic urine analysis had been performed and therefore only the eGFR could be considered to assess renal impairment. Data of other causes of renal impairment were lacking.

The strength of our study is its size of the patient pool. The number of patients included was limited by time constraints and human resources. The majority of persons had had only one creatinine measurement in 2011–2013, which does not meet the international recommendations to determine CKD. However, from side of the clinic there was neither a selective process preferring specific patients for laboratory tests, nor did the analysis yield a difference between patients with missing initial creatinine from the others with regard to age, sex and Hgb level. Individuals with a missing initial creatinine value had higher CD4 counts, indicating a less advanced immune deficiency. Hence, the true proportion of persons with moderately/severely impaired renal function might be closer to the full sample, and the results of the multivariable subgroup analyses slightly overestimate the effects.

Despite the limitations, we observed three distinct developments of renal function in this cohort: 1) some persons with initially normal or mildly impairment eGFR lost renal filtration over time, in addition to the aging effect. 2) persons whose renal function was initially severely impaired regained renal filtration most likely because of viral clearance. 3) a subgroup of individuals who were identified with impaired renal function deteriorated or remained at a low level, despite switching to a TDF-free regimen.

Our findings suggest that around one third of HIV patients in Zambia develop at least mildly impaired renal function. While renal monitoring would be recommended, it is not feasible in many parts of SSA and could slow down ART initiation, which contrasts the efforts to accelerate and expand ART access [11, 24]. We are well aware of the reassuring data of the DART trial showing non inferiority of long term outcomes in clinical driven monitoring compared to laboratory supported management. However, this trial was conducted before the broad introduction of TDF as first line treatment (up to Dec 2008) [25].

Increasing life expectancy, continuing population growth and increasing numbers of PLWH on lifelong ART contribute to higher incidence and prevalence of NCDs such as renal diseases. NCDs have been recognized as public health threat and already given priority in health policies in Zambia [26]. Recently launched first measures comprise training of NCD specialists in endocrinology and cardiology, and training of provincial NCD focal point persons at primary healthcare organizations, for instance. However, the persistent severe shortage of human resources for health services together with the lack of corresponding capacities prevent a broader transfer of policies to healthcare practice [27]. Given the fact of an anticipated healthcare workers gap of around 13,000 in 2020 in Zambia [28], a gradual integration of NCD diagnosis and treatment in HIV services, targeted renal function monitoring in patients at risk, and treatment of renal impairment could help to address this problem in resource limited settings. Innovative and flexible ART delivery approaches offered to well managed HIV patients might help to free resources for renal disease services. With respect to CKD screening, in contrast to routine lab-monitoring, a targeted approach based on a CKD risk score could be adapted to African populations and applied during treatment initiation [29]. Additionally, HIV programmes should consider the use of newer ARTs with less nephrotoxic effects such as integrase inhibitors and Tenofovir alafenamide (TAF), in particular if continuous renal monitoring is not feasible [30].

## Conclusions

Assessment of renal function in HIV cohorts in SSA remains a challenge. The use of ART seems to be associated with a decrease in eGFR over time. However, PLWH with moderately/severely impaired renal function prior to ART initiation appear to retain or improve their eGFR during ART. Further prospective cohorts are needed to improve the evidence pointing to a causal link. Furthermore, research should address adjusting eGFR equations according to the characteristics of SSA populations, and include HIV negative individuals to assess CKD burden in the general population. Targeted monitoring of renal function is recommended, however, should not delay access to ART nor hamper the delivery of HIV care.

## Supporting information

S1 Fig Individual ART periods of the HIV cohort (N = 1118) with last creatinine measurement between 2011 and end of 2013 at Chreso Ministries, Lusaka, Zambia. (JPEG)

S1 Table Anonymized dataset of 1118 HIV positive individuals on ART at Chreso Ministries, Lusaka, Zambia. (CSV)

## Abbreviations

ABC: abacavir
ALT: alanine aminotransferase
ART: antiretroviral treatment
AST: aspartate aminotransferase
BP: blood pressure
CKD: chronic kidney disease
CL: Chreso Lusaka
CM: Chreso Ministries
d4T: stavudine
EFV: efavirenz
eGFR: (estimated) glomerular filtration rate
FTC: emitricitabine
Hbg: haemoglobin
KDIGO: Kidney Disease Improving Global Outcomes
LPV: liponavir
MAP: mean arterial pressure
NVP: Nevirapine
NCD: non-communicable chronic diseases
PLWH: people living with HIV
rtv: ritonavir
SSA: sub-Saharan Africa
TDF: tenofovir/tenofovir disoproxile fumarate
