# Supplementary figures and images for: Assessment of renal function in routine care of people living with HIV on ART in a resource-limited setting in urban Zambia
Source: PLoS One. 2017 Sep 20;12(9):e0184766. doi: 10.1371/journal.pone.0184766 (PMC5607167; doi:10.1371/journal.pone.0184766)

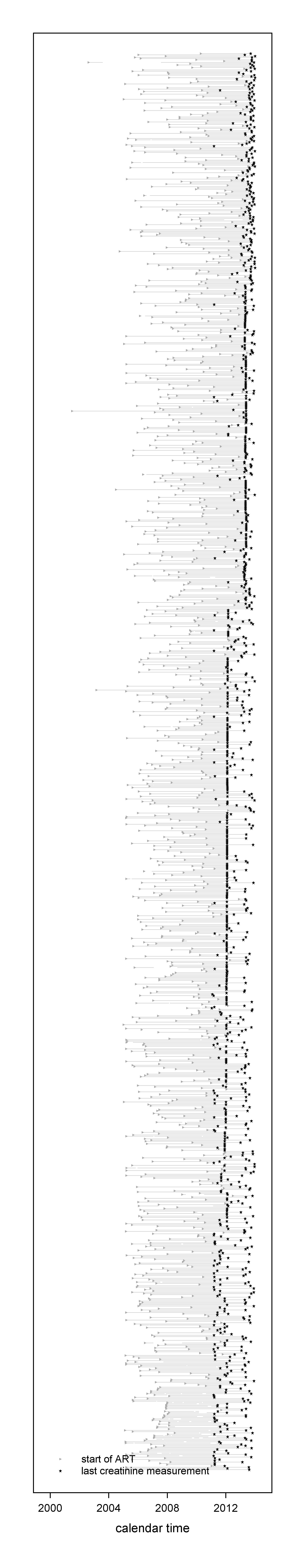

Supplement: S1 Fig — (JPEG) [file pone.0184766.s001.jpeg]
